# 

**Published:** 1883-05-20

**Authors:** 


### EDITORIALS AND MISCELLANEOUS.

### *EDITORIAL NOTICES.*

LAMBERT & Co.—See the new advertisement and Special Notice of this excellent house of St. Louis.

PORTABLE ELECTRIC LIGHT.—Read the advertisement of the Portable Electric Light Co.

A. L. HERNSTEIN.—Don't fail to see the advertisement of this house, commencing with this issue of our Journal.

SCOTT & BOWNE.—A new advertisement of this excellent establishment will be found in this issue of our Journal. Examine it carefully.

PRACTICE FOR SALE.—See this Card under Special Notice head. The location we know to be a good one. Letters addressed to this office in regard to it will meet with attention.

Dr. JOSEPH K. BARNES, ex-surgeon of the United States army, died of Bright's disease on April 5th, 1883, aged 66 years.

Prof. WM. H. VAN BUREN, of New York, died March 25th, 1883. His death was attributed to brain lesion, following an attack of apoplexy, which occurred in the spring of 1882. Dr. VanBuren was a surgeon of marked and growing celebrity.

MARYLAND MEDICAL JOURNAL.—From and after the May number of the present year, the Maryland Medical Journal will be issued as a weekly in the shape of a sixteen page double column publication. Success to brothers Ashley and Cordell, the able editors of this excellent journal.

### *PAMPHLETS RECEIVED.*

Illinois State Board of Health. Preventable-Disease Circulars.—Typhoid Fever, Diphtheria, etc.

Fifth Biennial Report of the Trustees, Superintendent and Treasurer of the Illinois Southern Hospital for the Insane, at Anna. H. Wardner, M. D., Superintendent. Oc ober 1, 1882.

Annual Address before the New York Medico-Chirurgical Society, by the President, Dr. E. P. Fowler. Delivered November 14th, 1882.

Annual Reports, 1882, of the Department of Health of the City of Charleston, South Carolina. Charleston, S. C.: The News and Courier Book Presses, 1883.

The Percentage of College-Bred Men in the Medical Profession, a paper read before the American Academy of Medicine, October 27th, 1882, by Charles McIntire, Jr., M. D., of Easton, Pennsylvania.

Suggestions regarding the local treatment of some of the commoner affections of the Ear, by Samuel Theobold, M. D., of Baltimore, Surgeon to the Baltimore Eye, Ear and Throat Charity Hospital, etc.

The Fifth Biennial Report of the Trustees, Superintendent and Treasurer of the Illinois Southern Hospital for the Insane, at Anna, October 1, '82. Springfield: H. W. Rokker, State Printer & Binder, 1883

---

### *RECEIPTED.*

(Receipts not acknowledged by letter, are recorded here.)

For 1882—Drs. J M Pierce, M F Alford, Wm Law, T D Hare, T L Appleby.

For 1883—Drs. J H Pool, Jno Hardamon, J B Lee, E M Pharr, Z T Young, T H Lyon, E Wheeler, J Dillworth, R L Foreman, T L Quillian, T D Hare, J M Underwood.

